# Transcriptome Analysis Coupled with Metabolome Profiling at a Key Time Point Reveals the Molecular Mechanism of Cold Stress Response in Oil Palm (*Elaeis guineensis* Jacq.)

**DOI:** 10.3390/plants15111628

**Published:** 2026-05-26

**Authors:** Qiufei Wu, Zhihao Zhao, Zongming Li, Rui Li, Xianhai Zeng, Lixia Zhou

**Affiliations:** 1 State Key Laboratory of Tropical Crop Breeding, Coconut Research Institute, Chinese Academy of Tropical Agricultural Sciences, Sanya 572025, China; qfi_wu@catas.cn (Q.W.); zzh5268@163.com (Z.Z.); zmli@catas.cn (Z.L.); lirui@catas.cn (R.L.); xhzeng@126.com (X.Z.); 2 Industrial Development Department, Chinese Academy of Tropical Agricultural Sciences, Haikou 571101, China

**Keywords:** oil palm (*Elaeis guineensis* Jacq.), cold stress, RNA-seq, metabolomics, WGCNA

## Abstract

Cold stress poses a major threat to global agricultural productivity. As a tropical woody oil crop, oil palm is highly susceptible to chilling damage; however, the molecular mechanisms underlying its cold response remain largely unknown. In this study, we profiled spear leaves of oil palm seedlings exposed to 8 °C for 0, 0.5, 1, 2, 4 and 8 h, using transcriptomic analysis across the full time course, complemented by metabolomic profiling at the 2 h time point. Physiological measurements showed cold stress-associated changes in chlorophyll and malondialdehyde (MDA) levels, as well as in the activities of antioxidant enzymes (SOD, POD, and CAT). Transcriptome analysis identified 31,576 expressed genes, including 9042 differentially expressed genes (DEGs). The highest number of specific DEGs was observed at the 2 h time point. Weighted gene co-expression network analysis (WGCNA) revealed nine co-expression modules with distinct temporal patterns. A total of 46 hub genes were identified, including WRKY, ERF, and seven genes encoding key enzymes involved in the biosynthesis of phenylalanine, tyrosine, and tryptophan (*LOC105041937*, *LOC105056784*, *LOC105048637*, *LOC105055093*, *LOC105038203*, *LOC105033050*, and *LOC105037948*). Metabolomic analysis detected 98 differentially accumulated metabolites, which were enriched in the phenylalanine, tyrosine, and tryptophan pathway. qRT-PCR analysis showed that *WRKY* and *ERF* expression peaked at 2 h, coinciding with phenylalanine accumulation. In summary, this study describes the temporal dynamics of the cold stress response in oil palm, identifies the 2 h time point as a transition period, and provides a set of prioritized hub genes for further functional validation. These findings may support future breeding efforts aimed at improving cold tolerance in oil palm.

## 1. Introduction

Oil palm (*Elaeis guineensis* Jacq.) is the tropical woody oil crop with the highest oil yield in the world [1]. Originating from tropical Africa, it is mainly distributed between 10° S and 15° N in tropical countries and regions of Asia, Africa, and Latin America. This species thrives under conditions of high temperature, abundant rainfall, and intense sunlight, with an optimal growth temperature ranging from 24 to 27 °C [2,3]. Growth is adversely affected when temperatures drop below 15 °C. Oil palm was introduced to China in the 1930s and is now mainly cultivated in Hainan, Yunnan, Guangdong, Guangxi, and Fujian, roughly between 98° and 118° E, and 18° –25° N. With the exception of southern Hainan Island, other tropical areas in China lie on the margins of the tropical zone [4]. In these regions, the annual average temperature ranges from 19.6 °C to 25.6 °C, which is considerably lower than that of the native range of the oil palm. China currently relies entirely on imported palm oil and is the world’s second-largest importer. Tropical regions in China frequently experience cold events, and extreme low temperatures in particular pose a serious threat to the growth and development of oil palm [5]. Therefore, a comprehensive and in-depth investigation into the cold tolerance mechanisms of oil palm is urgently needed to promote the sustainable development of China’s oil palm industry. Previous studies have shown that short-term exposure to such conditions markedly reduces photosynthetic efficiency, membrane stability, and fatty acid desaturation, indicating a limited capacity for cold acclimation [6]. Despite its economic importance, the molecular mechanisms underlying cold stress responses in oil palm remain poorly understood. In this study, we performed an integrated physiological and transcriptomic analysis of oil palm leaves exposed to 8 °C across a time-course of 0.5–8 h, thereby capturing early transcriptional and physiological responses to chilling stress.

Plants are frequently subjected to various abiotic stresses, including the cold, heat, drought, heavy metals, and salt stress. These stresses restrict the geographical distribution of plants and reduce their productivity [7]. Among them, temperature plays a particularly decisive role in plant growth, as it is closely linked to the environmental temperature at every developmental stage [8]. Different varieties of the same species may have different temperature requirements. As the most common form of abiotic stress, temperature directly affects both yield and quality. Low-temperature stress can be divided into two main types based on the temperature range and severity. The first type, known as chilling, refers to temperatures between 0 and 15 °C. Chilling stress disrupts physiological and biochemical metabolism, slows down physiological activities, and ultimately reduces crop yield and fruit quality. The second type, known as freezing, refers to temperatures below 0 °C [9]. Severe freezing injury can cause cell dehydration and enzyme inactivation, preventing plants from carrying out normal physiological activities. This can lead to substantial economic losses, making freezing stress a typical natural disaster worldwide [10,11].

When exposed to cold stress, plants adjust their growth and development processes to better adapt to changes in the external environment. The reorganization of gene regulatory networks at the transcriptional level represents the most direct pathway for the identification and expression changes in cold-tolerance-related genes. By integrating RNA sequencing (RNA-seq) and chromatin immunoprecipitation sequencing (ChIP-seq) analyses, Lin et al. found that ICE1 directly altered the expression level of 76 downstream cold-responsive (COR) genes, which are likely closely associated with cold tolerance in tomato [12]. Using full-length transcriptome sequencing, Xiao et al. revealed that lncRNAs regulate cold tolerance in *P. canaliculata* by modulating the expression of genes involved in ubiquitin ligase E3, the 26S proteasome subunit non-ATPase, glutathione S-transferase, and cytochrome P450 [13]. However, plant responses to cold stress are a continuous process, and existing studies lack continuous tracking of transcriptional changes in oil palm under cold stress. The weighted gene co-expression network analysis (WGCNA) method is designed to identify modules of co-expressed genes and to explore the associations between gene networks and traits of interest, including the identification of core genes within the network. Applying WGCNA to transcriptome data enables the investigation of complex traits in plants during resistance to various environmental stresses, such as drought [14], cold [15], heat [16], oxidation [17], and salinity stress [18].

Metabolomics, which emerged after genomics and proteomics, has become an important component of systems biology [19]. Many life activities within cells occur at the metabolite level; processes such as cell signal release, energy transfer, and intercellular communication are all regulated by metabolites [20]. In particular, metabolomics can help reveal the complex mechanisms governing plant–environment interactions [21]. By studying metabolic pathways and their regulation, metabolomics can uncover the patterns of metabolite changes during metabolic processes [22]. Therefore, analyzing changes in plant metabolites under stress conditions using metabolomics and inferring the adjustments in metabolic pathways that plants employ in response to stress provides a powerful analytical approach for investigating the mechanisms underlying plant stress tolerance and adaptation.

In recent years, a range of metabolomics techniques have emerged, including liquid chromatography–tandem mass spectrometry (LC-MS), gas chromatography–mass spectrometry (GC-MS), liquid chromatography-ultraviolet detection (LC-UV), nuclear magnetic resonance (NMR), capillary electrophoresis mass spectrometry (CE-MS), and Fourier Transform Ion Cyclotron Resonance Mass Spectrometry (FTICR-MS). These advances have greatly promoted the development of metabolomics [23]. Many research groups have integrated transcriptomics with metabolomics to comprehensively analyze abiotic stress-responsive genes, metabolites, and metabolic pathways, aiming to elucidate the tolerance mechanisms of plants [24,25,26]. Nevertheless, the molecular mechanisms underlying cold stress responses remain poorly understood. In this study, transcriptomics combined with metabolomics was used to investigate the cold response of oil palm.

## 2. Materials and Methods

### 2.1. Cold Treatment and Sampling

Under normal conditions, African oil palm (*Elaeis guineensis*) seedlings were cultivated in nurseries. The seedlings were obtained from Wenchang, Hainan Province, China (9.63° N 110.94° E). All seedlings used for cold treatment were six-month-old nursery-grown plants raised under strictly controlled and uniform conditions. Specifically, the seedlings had an average height of approximately 45.37 cm and bore 6–8 expanded leaves. They were grown in nutrient soil with a pH of 5.6, composed of topsoil, coconut coir, organic fertilizer, and river sand at a ratio of 5:2:1:2 (*v*/*v*). Prior to treatment, the growth chamber conditions were maintained at 26 °C, with a light intensity of 1500–2000 lx, a photoperiod of 16 h light and 8 h dark, and a relative humidity of 60–70%. Following previously reported methods, the early key responses of oil palm to cold stress occur within 8 h after treatment [27,28]. Eighteen seedling plants that had germinated within the same week and were grown in the same nursery were selected for subsequent cold treatment. A group of these 18 seedlings was placed in a growth chamber at 26 °C for one day, after which three plants served as controls. The remaining 15 seedlings were subjected to 8 °C under a 16 h light/8 h dark cycle for 0.5, 1, 2, 4, and 8 h (Table S1). Control seedlings were maintained under a 16 h light/8 h dark cycle at 26 °C, with three biological replicates established for the cold treatment. Spear leaves were collected from both the control and cold-treated seedlings and immediately frozen in liquid nitrogen for RNA isolation.

### 2.2. Physiological Index Measurement

Changes in physiological and biochemical indices under cold stress were evaluated using fresh leaves harvested from the same set of six-month-old oil palm seedlings used for transcriptomic and metabolomic analyses. At each time point (0, 0.5, 1, 2, 4, and 8 h), spear leaves collected from each biological replicate were immediately divided: one portion was used fresh for the following assays, and the other was flash-frozen for omics analyses. The activities of superoxide dismutase (SOD), peroxidase (POD), and catalase (CAT) were measured [29,30]. In addition, a set of photosynthetic parameters, encompassing the chlorophyll a/b ratio, carotenoid/chlorophyll ratio, net photosynthetic rate (*Pn*), intercellular CO_2_ concentration (*Ci*), and stomatal conductance (*Gs*), were analyzed according to the cited methods [31,32].

### 2.3. RNA Extraction and RNA Sequencing

Total RNA was extracted from the flash-frozen leaf portions of the same biological replicates used for physiological measurements (see Section 2.2 for detailed sampling procedure). Specifically, leaf samples were collected from cold-treated and control oil palm plants at each time point, immediately frozen in liquid nitrogen, and stored at −80 °C until RNA extraction. RNA extraction was performed using a previously reported method [33]. First, the enriched mRNA was fragmented using a fragmentation buffer and then reverse-transcribed into first-strand cDNA with random primers. Second-strand cDNA was synthesized using DNA polymerase I, RNase H, dNTPs (with dUTP substituted for dTTP), and an appropriate reaction buffer. The resulting cDNA fragments were purified using the QiaQuick PCR extraction kit (Qiagen, Venlo, The Netherlands), followed by end repair, poly(A) tailing, and ligation to Illumina sequencing adapters. The second-strand cDNA was then digested with UNG (Uracil-N-Glycosylase). Finally, the digested products were size-selected by agarose gel electrophoresis, PCR-amplified, and sequenced on an Illumina HiSeq™ 4000 platform by Gene Denovo Biotechnology Co. (Guangzhou, China).

### 2.4. Transcriptome Data Analysis

Raw sequencing data were subjected to a stringent quality control (QC) process to produce high-quality clean reads. This involved filtering out adapter sequences, reads containing >10% unknown nucleotides (N), and low-quality reads where >50% of bases had a Q-score ≤ 20. The clean reads were then aligned to an rRNA database via Bowtie2 (v2.2.8) [34], and ribosomal RNA-derived reads were removed. To identify differentially expressed genes (DEGs) in response to cold stress, clean reads from each sample were aligned to the oil palm reference genome using HISAT2 (v2.1.0) [35]. Gene-level read counts were quantified using the prepDE. py script from the StringTie package (v1.3.4) [36]. Differential expression analysis was performed using the DESeq2 package with a significance threshold of |log_2_(fold change)| ≥ 1 and false discovery rate (FDR) < 0.05.

### 2.5. Co-Expression Network Analysis of Cold-Responsive Genes

Weighted gene co-expression network analysis (WGCNA) was performed on the transcriptome data to identify highly coordinated gene expression patterns across different stages of cold stress. To construct a robust co-expression network, genes with FPKM values greater than 5 were selected for module identification. This analysis was carried out using the AETVARE cloud platform (https://cloud.metware.cn/#/tools/tool-list, accessed on 16 May 2025).

### 2.6. Identification and Network Analysis of Hub Genes

Hub genes were screened based on all differentially expressed genes (DEGs) identified across the six time points (0, 0.5, 1, 2, 4, 8 h). Specifically, genes with the top 5% connectivity in the weighted gene co-expression network were designated as hub genes. These candidates were further validated using protein–protein interaction (PPI) networks (STRING database, confidence > 0.4, https://cn.string-db.org/) [37,38], and the CytoHubba plugin (Cytoscape v3.6.9) with 12 algorithms; genes recognized by ≥6 algorithms were selected as final hub genes [39].

### 2.7. Metabolite Extraction and Profiling Analysis

Following grinding in liquid nitrogen, 100 mg tissue samples were homogenized in a pre-chilled solution of 80% methanol and 0.1% formic acid. The homogenate was incubated on ice for 5 min and then centrifuged (15,000× *g*, 20 min, 4 °C). An aliquot of the supernatant was diluted with LC-MS grade water to a final concentration of 53% methanol. After a second centrifugation step under the same conditions, the final supernatant was injected into the LC-MS/MS system for analysis.

LC-MS/MS analysis was performed at Genedenovo (Guangzhou, China) using an ExionLC™ AD system (SCIEX) coupled with a QTRAP^®^ 6500+ mass spectrometer (SCIEX). Chromatographic separation was achieved on an Xselect HSS T3 column (2.1 × 150 mm, 2.5 µm) using a 20 min linear gradient at a flow rate of 0.4 mL/min. The mobile phases consisted of eluent A (0.1% formic acid in water) and eluent B (0.1% formic acid in acetonitrile) [40]. The gradient program was as follows: 2% B at 0–2 min, 2–100% B at 2–17 min, 100% B at 17–17.1 min, and 2% B at 17.1–20 min. The QTRAP^®^ 6500+ mass spectrometer was operated in both positive and negative electrospray ionization (ESI) modes. The source parameters common to both modes were: curtain gas, 35 psi; collision gas, medium; temperature, 550 °C; ion source gas 1, 60; ion source gas 2, 60. The ion spray voltage was set to 5500 V for positive mode and −4500 V for negative mode.

Metabolite detection was performed using Multiple Reaction Monitoring (MRM) with an in-house database. The Q3 ion was used for quantification, while a combination of Q1/Q3 ions, retention time (RT), declustering potential (DP), and collision energy (CE) was used for metabolite identification. Peak integration and correction were carried out in SCIEX OS version 1.4, with a minimum peak height of 500, a signal-to-noise ratio of 5, and a Gaussian smooth width of 1. The relative content of each metabolite was then calculated from its peak area.

### 2.8. Multivariate Statistical Analysis

For initial visualization of differences between sample groups, we performed principal component analysis (PCA), an unsupervised dimensionality reduction method, on all samples using the R package models (http://www.r-project.org/). PCA transforms hundreds of thousands of correlated metabolite variables into a smaller set of uncorrelated variables called principal components. To better distinguish metabolic profiles between two groups, we applied partial least squares discriminant analysis (PLS-DA), a supervised method that encodes class memberships as a matrix Y to identify variables correlated with group separation [41]. PLS-DA was performed on each comparison group using the R package ropls (http://www.r-project.org/) [42]. Variable importance in projection (VIP) scores from the (O)PLS model were used to rank the metabolites by their ability to discriminate between two groups, with a VIP threshold set to 1. In addition, we used the *t*-test as a univariate analysis to screen for differential metabolites. Metabolites with a *t*-test *p*-value < 0.05 and VIP ≥ 1 were considered differentially accumulated between two groups. Within each group, the abundance of differential metabolites was normalized using z-scores. VIP scores from OPLS-DA were then used for visualization. The top 15 metabolites are shown in descending order in the VIP score plot [43]. For pathway analysis, we used the Kyoto Encyclopedia of Genes and Genomes (KEGG), a major public database that includes both genes and metabolites [44]. Metabolites were mapped to KEGG metabolic pathways for annotation and enrichment analysis. Pathway enrichment analysis identified significantly enriched metabolic pathways or signal transduction pathways in differential metabolites compared with the whole background. The calculating formula is as follows:

P=1−∑i=0m−1 MiN−Mn−iNn

Here N is the number of all metabolites with KEGG annotation, n is the number of differential metabolites in N, M is the number of all metabolites annotated to specific pathways, and m is the number of differential metabolites in M. The calculated *p*-value went through FDR Correction, taking FDR ≤ 0.05 as a threshold. Pathways meeting this condition were defined as significantly enriched pathways in differential metabolites.

### 2.9. Real-Time Quantitative Reverse Transcription PCR (qRT-PCR) Analysis

To validate the RNA sequencing results, selected genes were analyzed using real-time quantitative PCR (qPCR). The RNA samples used for qRT-PCR were the same as those used for RNA-seq, which were derived from leaf tissues of three independent biological replicates under the same treatment conditions as described in Section 2.1. All reactions were carried out in a Mastercycler ep realplex4 system using 2 × SYBR Green qPCR ProMix (low ROX) in a 10 µL final volume on 96-well optical plates. The primers were listed in Table S2. The amplification protocol consisted of initial denaturation at 95 °C for 5 s, followed by 40 cycles of annealing at 55 °C for 15 s and extension at 68 °C for 20 s. A melting curve analysis was subsequently performed by gradually increasing the temperature from 60 °C to 95 °C over 20 min to assess reaction specificity. All assays included three biological and three technical replicates. Gene-specific primers are listed in Table S2. The ELF gene served as an internal control [45], and relative expression levels were calculated using the 2^−ΔΔCt^ method. Statistical significance (*p* < 0.05 and *p* < 0.01) was determined by one-way ANOVA using SPSS software.

### 2.10. Statistical Analyses

Statistical analyses were conducted with SPSS software (version 21.0). Significant differences were determined by analysis of variance (ANOVA), followed by Duncan’s multiple range test (DMRT) for post hoc comparisons at a *p*-value threshold of <0.05.

## 3. Results

### 3.1. Effects of Cold Stress on Physiological Indices of Oil Palm

**Figure 1:**
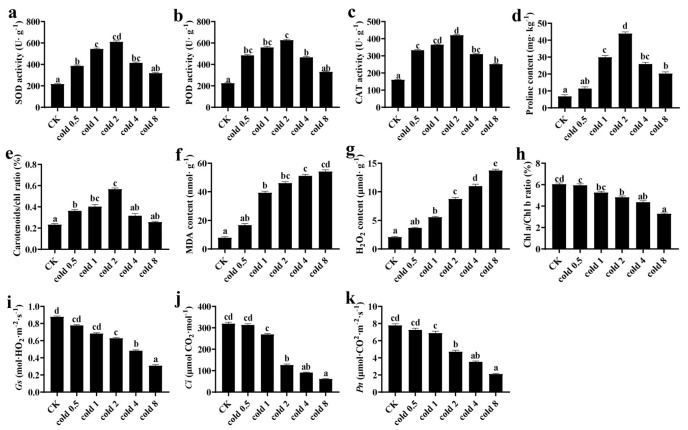
Physiological parameters, photosynthetic pigment contents, and gas exchange-related photosynthetic parameters measurement in oil palm leaves under cold conditions (0, 0.5, 1, 2, 4 and 8 h). (**a**) SOD activity (U·g^−1^), (**b**) POD activity (U·g^−1^), (**c**) CAT activity (U·g^−1^), (**d**) proline content (mg·kg^−1^), (**e**) carotenoid/chlorophyll ratio (%), (**f**) MDA content (nmol·g^−1^), (**g**) H_2_O_2_ content (μmol·g^−1^), (**h**) Chl a/Chl b (%), (**i**) stomatal conductance (*Gs*) (mol·HO_2_·m^−2^·s^−1^), (**j**) intercellular CO_2_ concentration (*Ci*) (μmol CO_2_·mol^−1^), and (**k**) net photosynthetic rate (*Pn*) (μmol·CO_2_·m^−2^·s^−1^). The letters on top of the figure bars follow the same analysis and modify the analysis statement in the figure legend (different lowercase letters indicate statistically significant differences among groups (*p* < 0.05) based on one-way ANOVA followed by Tukey’s post hoc test). Data are presented as mean ± standard deviation (SD) (*n* = 3).

In this study, significant physiological changes occurred in the fresh leaves of oil palm seedlings subjected to 8 °C stress for different durations (0, 0.5, 1, 2, 4, and 8 h). Minimal phenotypic changes were observed in oil palm seedlings after 0.5 and 1 h of cold stress relative to the 0 h baseline. At the 2 h time point, slight wilting occurred in some leaves. Following 4 h of stress, brown spots appeared on the leaves, suggesting the onset of chilling injury. By 8 h, these brown spots had spread across nearly the entire leaf surface, indicating severe cold damage (Figure S1). During the cold stress treatment, leaf thickness remained relatively unchanged throughout the experimental period. Regarding leaf width, no significant difference was observed between the cold-treated group and the control group at 0.5 h and 1 h, with average values of 4.058 cm and 4.017 cm, respectively. However, leaf width decreased to an average of 3.467 cm after 4 h of cold stress, followed by a more pronounced reduction to 3.061 cm at 8 h. In terms of chilling injury symptoms, no brown spots were detected on the leaf surface during the first 2 h of treatment. After 4 h, brown spots began to appear, with an average count of 3.333 per leaf, and this number increased significantly to 7 per leaf at 8 h (Table S3). The activities of superoxide dismutase (SOD), peroxidase (POD), and catalase (CAT) initially increased and then decreased as the duration of cold stress extended, peaking at the 2 h time point. At this peak, their levels were 2.82, 2.79, and 2.62 times those of the control group, respectively (Figure 1a−c). Proline content also reached its maximum after 2 h of cold stress, measuring 43.87 mg·kg^−1^, which was 6.48 times that of the control (Figure 1d). Similarly, the carotenoid/chlorophyll ratio peaked at 2 h of stress exposure (Figure 1e). In contrast, the contents of malondialdehyde (MDA) and hydrogen peroxide (H_2_O_2_) showed a gradual increase with prolonged stress duration (Figure 1f,g). Meanwhile, the Chl a/Chl b ratio (Figure 1h), stomatal conductance (*Gs*) (Figure 1i), intercellular CO_2_ concentration (*Ci*) (Figure 1j), and net photosynthetic rate (*Pn*) (Figure 1k) exhibited a continuous decline. These findings suggest that oil palm seedlings effectively mitigated cold stress within the first 2 h through a coordinated physiological response, including upregulation of the antioxidant enzyme system (SOD, POD, CAT), accumulation of the proline, and photosynthetic adjustments. However, under prolonged stress, oxidative damage intensified, as indicated by the accumulation of H_2_O_2_ and elevated MDA content (a marker of membrane lipid peroxidation). Concurrently, the photosynthetic system suffered irreversible damage, as evidenced by the decline in *Gs*, *Pn*, *Ci*, and the Chl a/Chl b ratio.

### 3.2. Analysis of Gene Expression in Oil Palm Leaves Under Cold Stress

Oil palm seedlings were subjected to cold treatment at 8 °C for six different durations (0, 0.5, 1, 2, 4, and 8 h). A total of 18 transcriptome libraries were constructed (Table S4). RNA sequencing generated over 1.60 billion raw reads, with an average of 88.95 million reads per library. After quality control, 99.69% of the reads (approximately 1.59 billion high-quality reads) were retained for subsequent analyses. On average, 92.65% of the clean reads were successfully mapped to the oil palm reference genome; detailed sequencing and mapping statistics are provided in Table S1. Gene expression levels were quantified using FPKM (fragments per kilobase of transcript per million mapped reads), leading to the identification of 3157 expressed genes. Violin plots depicting the distribution of gene expression levels across samples showed generally comparable expression profiles among the different time-point groups, as well as good reproducibility across biological replicates (Figure S2a). Correlation analysis further supported these findings: correlation coefficients between replicates within the same group exceeded 0.9, while those between different cold treatment groups ranged from 0.8 to 0.9. In contrast, correlations between the cold-treated samples and the control group ranged from 0.5 to 0.7, indicating that cold stress induced substantial transcriptomic changes in oil palm (Figure S2b).

**Figure 2:**
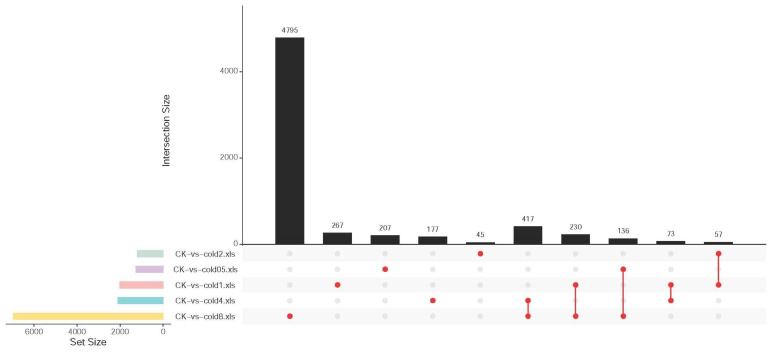
Distribution of DEGs in oil palm leaf treated with cold stress. Red dots denote the highest number of specific DEGs, while lines indicate differentially expressed genes.

Analysis of differentially expressed genes (DEGs) across the cold treatment time course revealed that each duration possessed a unique transcriptional signature. Notably, the 2 h time point induced the highest number of specific DEGs in leaves (Figure 2). Furthermore, a core set of 1739 DEGs was common to all four treatment durations, suggesting their central importance in the cold stress response. Expression pattern analysis indicated that the majority of these core DEGs were either continuously up-regulated or down-regulated throughout the stress period (Figure S3). Gene ontology (GO) enrichment analysis categorized these leaf DEGs primarily into broad biological processes, including “cellular process”, “metabolic process”, and “biological regulation” (Figure S4).

### 3.3. Weighted Gene Co-Expression Network Analysis (WGCNA)

A total of 9042 differentially expressed genes (DEGs) were identified using DESeq2 with a threshold of |log_2_(fold change)| ≥ 1 and FDR < 0.05. After filtering out genes with low expression levels (FPKM ≤ 5) across all samples, the remaining DEGs were used for co-expression network analysis. Co-expression modules were constructed based on the expression profiles of these DEGs. Hierarchical clustering of samples performed using the flashClust package in the WGCNA algorithm showed no outlier samples (Figure S5a). The topological overlap measure (TOM) among co-expressed genes across different modules is presented as a heatmap in Figure S5b. Ultimately, nine co-expression modules were established in the leaf co-expression network, with no gray module detected. The gene composition of each module is detailed in Table S4.

**Figure 3:**
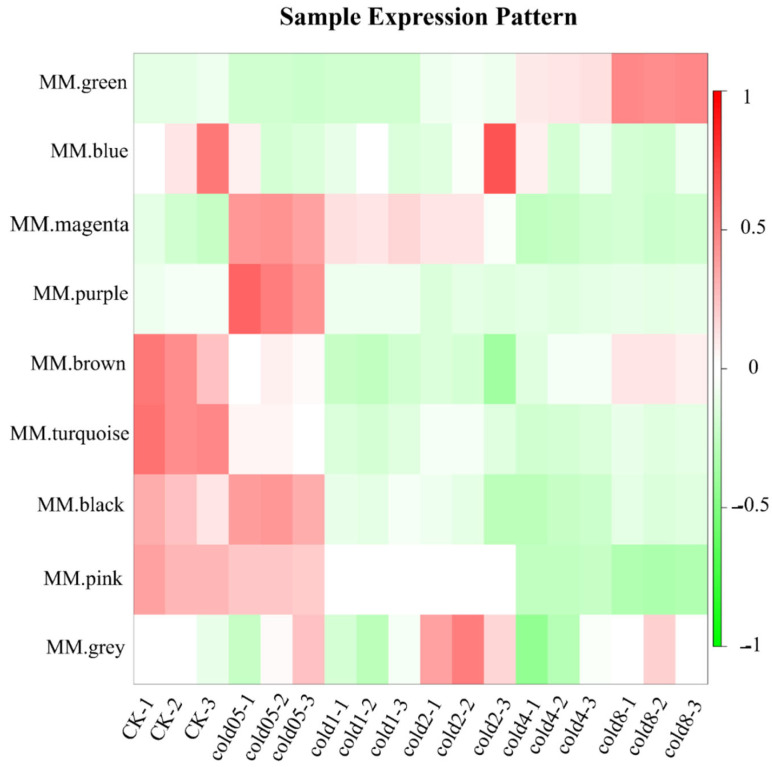
Heatmap displaying the correlation strength of each gene co-expression module with leaf samples.

We subsequently investigated how the nine identified modules correlated with the intensity (duration) of cold stress (Figure 3). Analysis revealed that genes within key modules exhibited pronounced differential expression precisely at the time points with which their modules were most strongly associated (Figure S6). Under cold stress, the modules displayed distinct temporal expression dynamics: The green module was markedly up-regulated only at the 8 h mark, in contrast to the general down-regulation observed at earlier points. The turquoise module was predominantly suppressed, showing significant down-regulation. The purple module displayed a biphasic response, with a subset of genes rapidly induced after 0.5 h, while the majority were repressed across other treatment durations.

**Figure 4:**
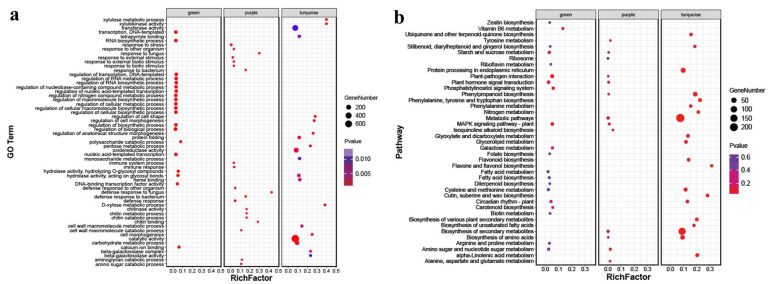
Enrichment analysis of critical modules. (**a**) GO terms, (**b**) KEGG pathways.

GO enrichment analysis revealed distinct functional specializations among the co-expression modules (Figure 4a). In the biological process category, the green module was significantly enriched for general stress responses (e.g., response to stress, GO:0006950 and defense response, GO:0006952), whereas the purple module was specifically associated with fungal defense (e.g., response to fungus, GO:0009620 and defense response to fungus, GO:0050832). In contrast, the turquoise module was primarily involved in xylulose metabolic (GO:0005997) and D-xylose metabolic processes (GO:0042732). Notably, the beta-galactosidase complex (GO:0009341) was the most significantly enriched cellular component term across these three modules. Molecular function analysis further highlighted their divergent roles: the green module was enriched in glycosyl hydrolase activities (GO:0004553, GO:0016798), the purple module in chitin-related functions (GO:0008061, GO:0004568), and the turquoise module in xylulokinase and heme binding activities (GO:0004856, GO:0020037) (Figure 4a).

KEGG enrichment analysis further delineated the functional identities of the key modules (Figure 4b). The transcriptional signature of the green module converged on stress perception and signaling, being substantially enriched in pathways such as plant–pathogen interaction, the MAPK signaling pathway, and the phosphatidylinositol signaling system. On the other hand, the purple module’s expression profile centered on metabolic biosynthesis, showing significant enrichment in the biosynthesis of isoquinoline alkaloids and the metabolism of amino sugars and nucleotides.

### 3.4. Identification and Expression Analysis of Hub Genes

**Figure 5:**
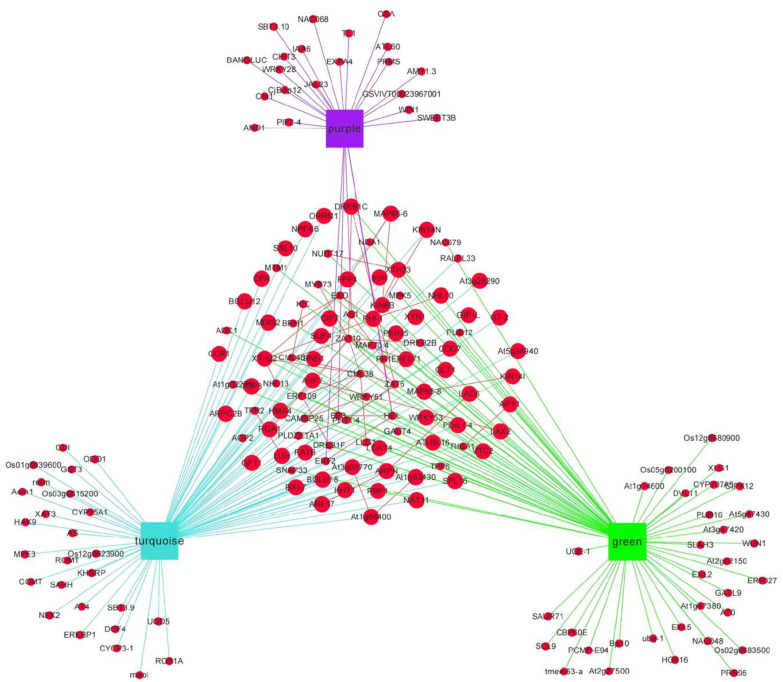
The architecture of the cold stress-responsive transcriptional network in oil palm. This network illustrates hub genes (large circles) and other DEGs (small circles), with red and green coloring indicating up- and down-regulation, respectively. Genes are clustered by their respective co-expression modules (squares). The connections (edges) between nodes encode two types of relationships: protein–protein interactions (red lines, from STRING database) and gene–module affiliations (other colored lines).

To identify key regulatory genes, we defined hub genes as the most highly interconnected genes within each co-expression module. This analysis, combining intramodular connectivity and CytoHubba assessment, identified 46 hub genes pertinent to cold stress (Table S5). These 46 hub genes represent candidate key regulatory factors with varying levels of evidential support. Among them, 27 genes demonstrate strong homology to known cold-responsive genes in model plants and are supported by evidence from the literature, suggesting they are high-priority targets for further functional validation (Table S6). A subset of these hub genes were transcription factors, such as WRKY, ERF, and ARF. Integrating these hub genes with the DEGs enabled the construction of a comprehensive cold stress response transcriptional network for oil palm leaves (Figure 5). Furthermore, potential interactions among the proteins encoded by these genes were annotated using the STRING database.

### 3.5. Validation of RNA-seq Data by qRT-PCR

**Figure 6:**
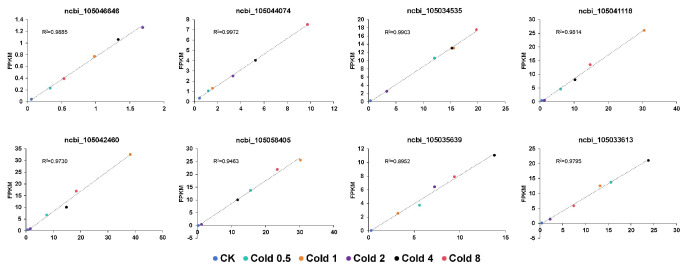
Correlation analysis of hub gene expression. Expression values from qRT-PCR (2^−ΔΔCt^, X-axis) and RNA-seq (FPKM, Y-axis) under cold stress are shown. Data points represent mean values from three biological replicates.

To validate the RNA-seq data, expression levels of eight randomly selected hub genes were measured by qRT-PCR. A strong positive correlation (R^2^ > 0.9 for seven out of eight genes) between qRT-PCR and RNA-seq (FPKM) data confirms the high reliability of the transcriptome analysis (Figure 6). These results indicated the high reliability of high-throughput sequencing.

### 3.6. The Response of the Oil Palm Metabolome to Cold Stress

**Figure 7:**
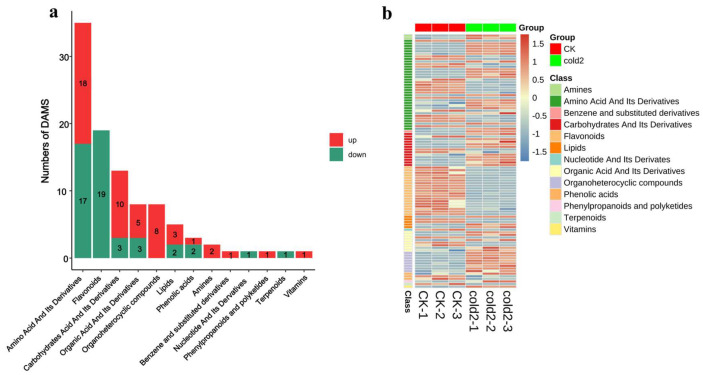
Differentially accumulated metabolites (DAMs) in oil palm leaves under cold stress (**a**) and DAMs in CK and Cold 2 samples of oil palm leaves (**b**). The heatmap displays the accumulation patterns of all identified DAMs across three biological replicates per treatment group.

Based on the maximal physiological and transcriptional disruption observed at 2 h, this time point was chosen for metabolic investigation. Untargeted metabolomics of oil palm leaves showed a significant metabolite shift under cold stress, evidenced by a 64.3% total variation in PCA scores (Figure S7a). The robustness of the PLS-DA model, confirmed by a permutation test (Figure S7b), supported the high confidence in identifying 98 differentially accumulated metabolites (DAMs). These DAMs, distributed across 13 super-classes, were almost equally split between up- (50) and down-regulation (48) (Figure 7a, Tables S7 and S8). As shown in Table S7, the most abundant super-classes were Amino Acids and Their Derivatives (35 DAMs, 35.7% of total), followed by Flavonoids (19 DAMs, 19.4%) and Carbohydrates and Derivatives (12 DAMs, 12.2%). Other super-classes included Organic Acids and Derivatives (8), Organoheterocyclic compounds (8), Lipids (5), Phenolic acids (3), Amines (2), and five additional classes with one DAM each (Benzene derivatives, Nucleotides, Phenylpropanoids, Terpenoids, and Vitamins). Consistent with the enrichment of the Carbohydrates and Derivatives category, we observed increased levels of D-glucose 6-phosphate and D-fructose 6-phosphate following cold stress (Table S6). Pathway analysis highlighted a significant enrichment in the phenylalanine, tyrosine, and tryptophan biosynthesis pathway (ko00400) (Figure S8, Table S9).

**Figure 8:**
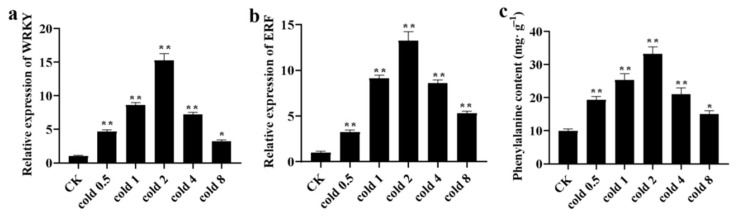
Expression of WRKR and ERF and phenylalanine content under cold stress. Data are presented as mean ± SD from three independent biological replicates. Statistical significance was assessed using Student’s *t*-test (* *p* < 0.05, ** *p* < 0.01). (**a**) Relative expression of WRKY; (**b**) Relative expression of ERF. (**c**) Phenylalanine content.

To validate the regulatory relationship between hub genes and key metabolites, we measured WRKY and ERF expression by qRT-PCR and phenylalanine content across the cold stress time course (0–8 h). Both genes increased from 0.5 to 2 h and peaked at 2 h (WRKY: 15.26, 14.21-fold over control; ERF: 10.60, 9.49-fold over control) (Figure 8a,b). Phenylalanine followed the same trend, peaking at 2 h (33.26 mg·g^−1^, 2.34-fold over control) (Figure 8c). These results support the regulatory network.

### 3.7. DEG and DAM Profiles in Cold-Stressed Oil Palm

**Figure 9:**
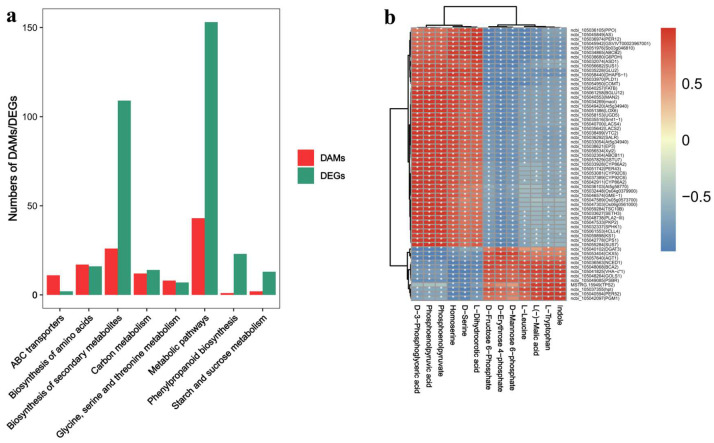
Multi-omics integration of cold stress responses. (**a**) Co-enriched KEGG pathways from DEG and DAM profiling. (**b**) Gene–metabolite correlation heatmap. Asterisks represent *p *≤ 0.05.

To elucidate the interplay between transcriptional and metabolic reprogramming, we performed a joint KEGG enrichment analysis, which identified eight co-enriched pathways significantly affected by cold stress (Figure 9a). Furthermore, we constructed a regulatory network based on correlation analysis between DEGs and DAMs. The heatmap in Figure 9b presents the top 61 most significant Spearman’s correlation pairs, highlighting the strongest putative gene–metabolite interactions.

### 3.8. Expression Dynamics of Phenylalanine Biosynthesis Pathway Genes Under Cold Stress

**Figure 10:**
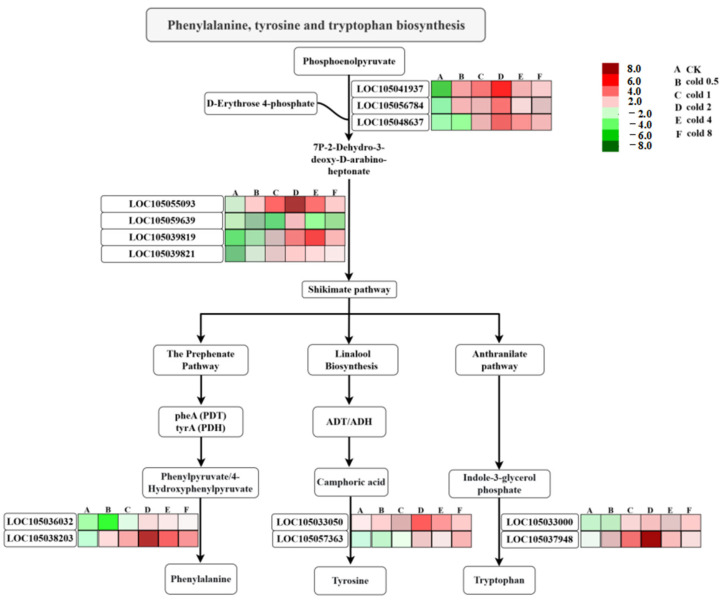
Heatmap visualization of gene expression dynamics in phenylalanine, tyrosine and tryptophan biosynthesis. Red and green hues denote up- and down-regulated DEGs, respectively, based on FPKM values.

To understand how phenylalanine accumulation is regulated at the transcriptional level, we examined the expression of key genes in the phenylalanine, tyrosine, and tryptophan biosynthesis pathway (KEGG ko00400). Analysis of our transcriptome data (based on KEGG annotation egu00400) showed that most genes in this pathway were progressively up-regulated from 0.5 to 2 h of cold stress (*LOC105041937*, *LOC105056784*, *LOC105048637*, *LOC105055093*, *LOC105038203*, *LOC105033050* and *LOC105037948*). Their expression peaked at the 2 h time point and then declined at 4 and 8 h (Figure 10). This expression pattern was consistently observed across all branches of the pathway: the shikimate pathway (M00022), the phenylalanine-specific branch (M00910), and the tyrosine (M00040) and tryptophan (M00023) branches.

## 4. Discussion

As a key tropical oil crop with the highest oil production efficiency worldwide, oil palm faces a major constraint in China: low winter temperatures in southern cultivation areas restrict its growth and yield, thereby hindering industrial development [46]. Elucidating the molecular mechanisms underlying oil palm responses to cold stress is therefore crucial for developing cold-tolerant varieties and expanding cultivation into colder climates [47,48]. Multi-omics approaches integrated with high-throughput sequencing offer powerful strategies to systematically unravel the complex molecular networks involved in plant stress adaptation [49,50]. In this study, we employed integrated transcriptomic and metabolomic analyses to investigate the dynamic response of oil palm to cold stress, with a particular focus on identifying time-specific regulatory networks.

### 4.1. The 2 h Time Point Represents a Critical Threshold in the Cold Stress Response

Cold stress typically induces a growth lag phase in plants, accompanied by decreased leaf relative water content and osmotic stress [51,52]. In response, plants synthesize and accumulate osmoprotectants to maintain cellular homeostasis [53]. Our observation of increased D-glucose 6-phosphate and D-fructose 6-phosphate following cold stress (Table S6) is consistent with well-established roles of soluble sugars as osmoprotectants and ROS scavengers in various plant species, including coconut [54], *Cucurbita pepo* [55], and Chinese cabbage [56]. This suggests that sugar-mediated osmotic adjustment represents an evolutionarily conserved defense mechanism also operative in oil palm.

Physiologically, the 2 h time point exhibited peak activities of key antioxidant enzymes (SOD, POD, CAT) and maximal proline accumulation (Figure 1a–d). Similar temporal patterns have been reported in cold-responsive species such as *Digitalis purpurea* and *Digitalis purpurea*, where antioxidant activity peaks within the first few hours of stress before declining due to oxidative damage accumulation [57,58]. This coordinated upsurge reflects an effective initial defense aimed at scavenging ROS and preserving osmotic homeostasis. Notably, the 2 h treatment triggered the highest number of unique DEGs (Figure 2), a pattern comparable to that observed in cold-treated bean, where early time points (24 h) show the most pronounced transcriptional activation [59]. The subsequent decline in antioxidant enzyme activities coupled with progressive increases in H_2_O_2_ and MDA beyond this time point (Figure 1f,g) indicates that cellular defense capacity becomes overwhelmed, leading to oxidative damage and membrane lipid peroxidation.

### 4.2. Transcriptional Networks and Hub Genes Reveal the Regulatory Architecture of Cold Tolerance

Weighted gene co-expression network analysis (WGCNA) has been successfully able to resolve functional specialization underlying transcriptional responses to abiotic stresses in multiple plant species, including quinoa and rapeseed [60,61]. Applying WGCNA to oil palm cold stress data, we identified distinct co-expression modules associated with general stress responses (green module), fungal defense (purple module), and sugar metabolism (turquoise module) (Figure 4). The enrichment of the green module in plant–pathogen interaction and MAPK signaling pathways (Figure 4b) aligns with growing evidence of crosstalk between abiotic and biotic stress signaling pathways. Similar observations in rice show that cold stress activates defense-related genes typically associated with pathogen response, suggesting an evolutionary integration of stress signaling networks [62].

Our identification of 46 hub genes, including WRKY, ERF, and ARF transcription factors (Table S5, Figure 5), provides a valuable set of candidates for functional validation. WRKY factors are known regulators of cold tolerance; for instance, *PmWRKY57* from *Prunus mume* improves cold tolerance in *Arabidopsis thaliana* [63]. The *OsWRKY63*-*OsWRKY76*-*OsDREB1B* module regulates chilling tolerance in rice [64]. ERF family members, especially the C-repeat Binding Factors (CBFs), serve as master regulators of cold acclimation across angiosperms, from *Arabidopsis* to temperate grasses and woody perennials (e.g., oil palm, rubber tree and cassava) [65,66,67,68,69]. The presence of these conserved families among our hub genes points to a regulatory architecture in oil palm that mirrors that of other cold-responsive plants, although individual isoform functions may be distinct. The strong concordance (R^2^ > 0.9) between RNA-seq and qRT-PCR results (Figure 6) confirms the reliability of our transcriptomic dataset and substantiates the inferred regulatory network.

### 4.3. Integrated Metabolomic and Transcriptomic Analyses Uncover Key Biochemical Pathways

Integrating transcriptomic and metabolomic data provides a systems-level view of stress responses that neither technique can achieve independently [70]. Our observation of a pronounced metabolomic shift at the 2 h stress point (Figure 7a), coupled with 98 differentially accumulated metabolites (DAMs), demonstrates that transcriptional reprogramming at this critical juncture translates into functional metabolic changes. Notably, the marked enrichment of the phenylalanine, tyrosine, and tryptophan biosynthesis pathway (Figure 8a) echoes findings from cold stress studies in *Tartary Buckwheat*, soybean, and tea, where this pathway serves as a central hub for producing flavonoids, lignin, and indole alkaloids [71,72,73]. These specialized metabolites function as antioxidants, UV screens, and cell wall structural components during abiotic stress adaptation [74,75].

Our transcriptome data further showed that most genes in this pathway (ko00400) were coordinately up-regulated during early cold stress. Their expression peaked at 2 h and then declined (Figure 10). This pattern was consistent across all pathway branches: the shikimate pathway (M00022), the phenylalanine-specific branch (M00910), and the tyrosine (M00040) and tryptophan (M00023) branches. This coordinated expression coincided with the maximum accumulation of phenylalanine at 2 h (33.26 mg·g^−1^, 2.34-fold over control). The early induction of this pathway is consistent with the known role of aromatic amino acids in plant stress defense. Phenylalanine is a precursor for flavonoids, lignin, and phenolic acids, which contribute to antioxidant capacity and cell wall strengthening under stress conditions [76]. The decline in both gene expression and phenylalanine content after 2 h may result from feedback inhibition by accumulated metabolites. It may also reflect a general suppression of primary metabolism due to prolonged stress-induced damage, such as reduced photosynthesis and increased oxidative stress (Section 3.1).

### 4.4. A Proposed Model for the Cold Stress Response in Oil Palm

Based on our integrated multi-omics data and comparisons with existing literature, we propose a coordinated defense model in which oil palm seedlings rapidly activate a protective transcriptional program upon cold exposure (Figure 9). Key hub transcription factors, particularly WRKYs and ERFs, are promptly induced and orchestrate the up-regulation of: (1) The antioxidant enzyme system (SOD, POD, CAT), which directly scavenges ROS bursts [77]; (2) osmoprotectant synthesis pathways facilitating proline and soluble sugar accumulation (e.g., D-glucose 6-phosphate), thereby preserving cellular integrity and water homeostasis [78], and (3) phenylpropanoid and other secondary metabolite pathways that generate protective compounds to mitigate oxidative and cellular damage [79]. This model shares core features with the cold stress response paradigms established in sugar beet, alfalfa, and other crops, namely, rapid antioxidant activation, osmolyte accumulation, and reprogramming of secondary metabolism [50,80].

This coordinated defense response peaks at approximately 2 h, reflecting the oil palm’s maximal effort to maintain cellular homeostasis. However, as cold stress persists beyond this critical window, sustained energy expenditure and accumulating damage lead to a marked suppression of primary metabolic processes, including photosynthesis, as indicated by declining in *Pn*, *Gs*, and *Ci* (Figure 1i–k). This eventual collapse of the defense system is consistent with observations in chill-sensitive species like cucumber and tomato, where prolonged cold exposure overwhelms initial protective responses and leads to irreversible damage [50].

### 4.5. Limitations of the Study

It should be noted that this study has several experimental limitations. First, only three biological replicates were included per time point, and all 18 seedlings originated from a single batch, which limits the assessment of individual variation and batch-to-batch variability. Second, all cold treatment durations (e.g., 0.5 h and 8 h) shared a common 0 h control, with no independent, time-matched controls maintained at 26 °C for each time point. Consequently, the observed changes in gene expression or metabolite levels cannot be attributed solely to cold stress, as potential confounding effects from circadian rhythms or other time-dependent factors cannot be fully excluded. Although this study primarily focused on short-term cold responses, the interpretation of dynamic changes between 0.5 h and 8 h should be considered in light of this design constraint. Third, while transcriptomic analysis covered six time points (0, 0.5, 1, 2, 4, and 8 h), metabolomic analysis was limited to the 2 h time point. As noted by a reviewer, this disparity limits the depth of an “integrated” multi-omics interpretation. Although describing our approach as “integrated transcriptomic and metabolomic analysis” remains technically accurate for the available 2 h metabolomic data, we acknowledge that full temporal integration was not achieved. Future studies should incorporate time-matched control samples to better dissect the effects of cold stress from time-related factors and should also include metabolomic data from additional time points (e.g., 0 h control and 8 h) to enable complete time-series integration.

## 5. Conclusions

This study delineates the temporal dynamics and interconnected molecular networks governing the cold stress response in oil palm. We identify the 2 h post-stress period as a critical physiological transition point. Our network analysis prioritizes a set of hub genes, including transcription factors such as WRKY, and ERF, along with key enzymes in the phenylpropanoid pathway (*LOC105041937*, *LOC105056784*, *LOC105048637*, *LOC105055093*, *LOC105038203*, *LOC105033050* and *LOC105037948*), as high-confidence targets. Given their significant differential expression and central network connectivity, we propose these genes as prioritized candidates for marker-assisted selection or gene editing. Moreover, the substantial reprogramming of secondary metabolism highlights its essential role in cold adaptation. Collectively, these findings provide a shortlist of actionable genetic targets, establishing a precise foundation for breeding strategies aimed at enhancing cold tolerance in this economically vital crop.

## Data Availability

The original contributions presented in this study are included in the article/Supplementary Material. Further inquiries can be directed to the corresponding author.

## Supplementary Materials

The following supporting information can be downloaded at https://www.mdpi.com/article/10.3390/plants15111628/s1. Figure S1. Growth phenotypes of seedlings from different treatment groups after the stress period. Briefly, 1–3 means three biological replicates. Figure S2. Overview of gene expression patterns in leaf samples under cold stress. (a) Expression level bar plot. (b) Gene correlation heatmap. Figure S3. Expression patterns of DEGs in oil palm leaves. Figure S4. GO annotation of DEGs in oil palm leaves. Figure S5. Gene co-expression network analysis in oil palm leaves. (a) Outlier detection. (b) TOM heatmap depicting hierarchical clustering of co-expressed genes. Figure S6. Heatmap showing the expression profiles of genes in a key module from oil palm leaves. Figure S7. Principal component analysis (PCA) and partial least squares discrimination analysis (PLS-DA) of metabolites from CK and cold-treated plants. (a) PCA score plot. (b) PLS-DA score plot. Figure S8. KEGG annotation of DEMs in oil palm leaves. Supplementary Table S1. Overview of cold treatment duration and sampling time. Supplementary Table S2. Primers for qPCR. Supplementary Table S3. Time-course changes in leaf width and brown spot count of oil palm leaves under cold stress treatment. Supplementary Table S4. Summary of RNA-seq data. Supplementary Table S5. Details information on differentially expressed genes (DEGs) in leaves under cold stress. Supplementary Table S6. The hub genes detected in WGCNA modules in oil palm leaves [81,82,83,84,85,86,87,88,89,90,91,92,93,94,95,96,97,98,99,100,101,102]. Supplementary Table S7. Details information on differentially accumulated metabolites (DAMs) in leaves under cold stress. Supplementary Table S8. Details information on differentially accumulated metabolites (DAMs) in oil palm leaves under cold stress. Supplementary Table S9. KEGG analysis of differentially accumulated metabolites (DAMs) in oil palm leaves under cold stress.
