# Supplementary figures and images for: Transcriptome Analysis Coupled with Metabolome Profiling at a Key Time Point Reveals the Molecular Mechanism of Cold Stress Response in Oil Palm (Elaeis guineensis Jacq.)
Source: Plants (Basel). 2026 May 26;15(11):1628. doi: 10.3390/plants15111628 (PMC13258973; doi:10.3390/plants15111628)

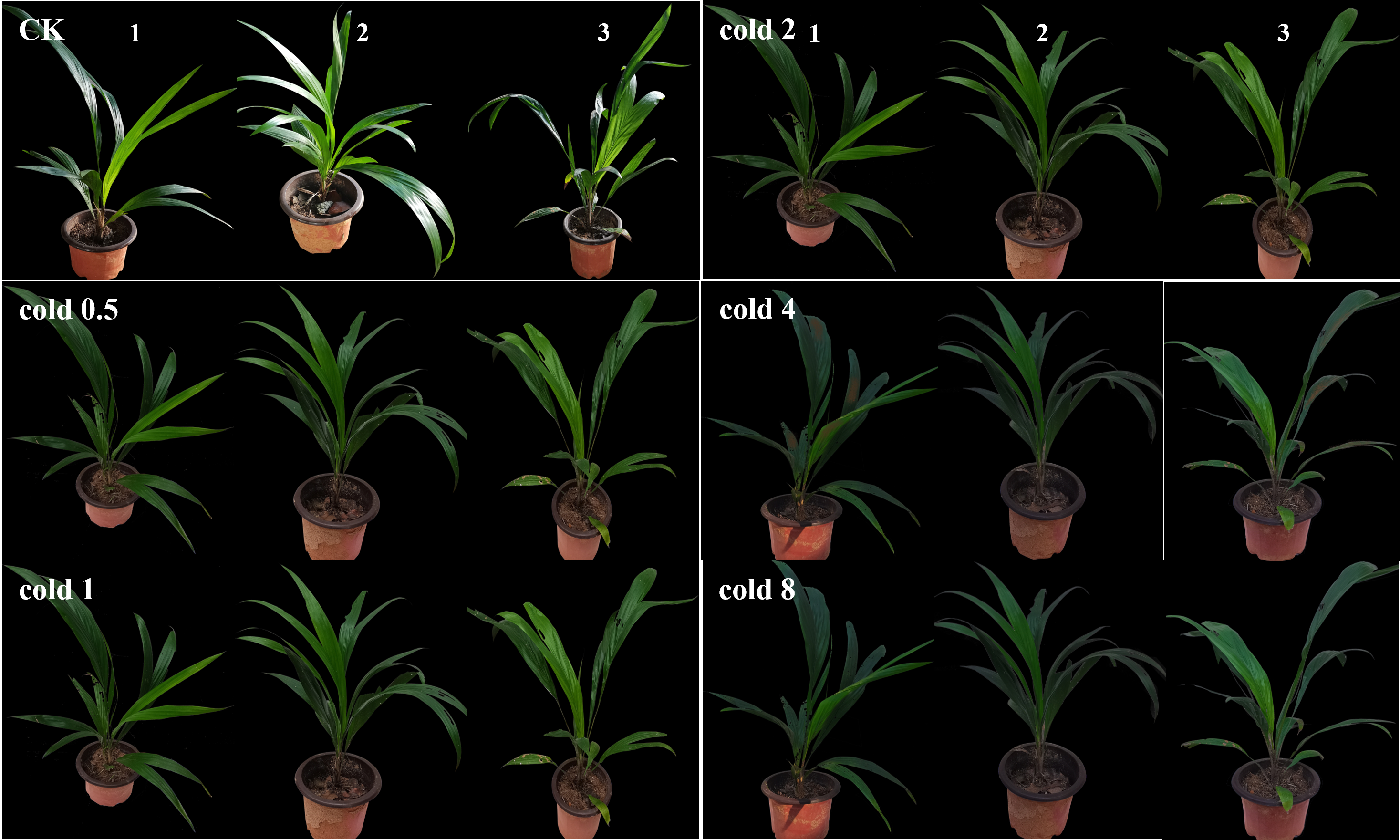

Supplement: Supplementary file 1 [file plants-15-01628-s001.zip › Supplemental Figure S1.png]

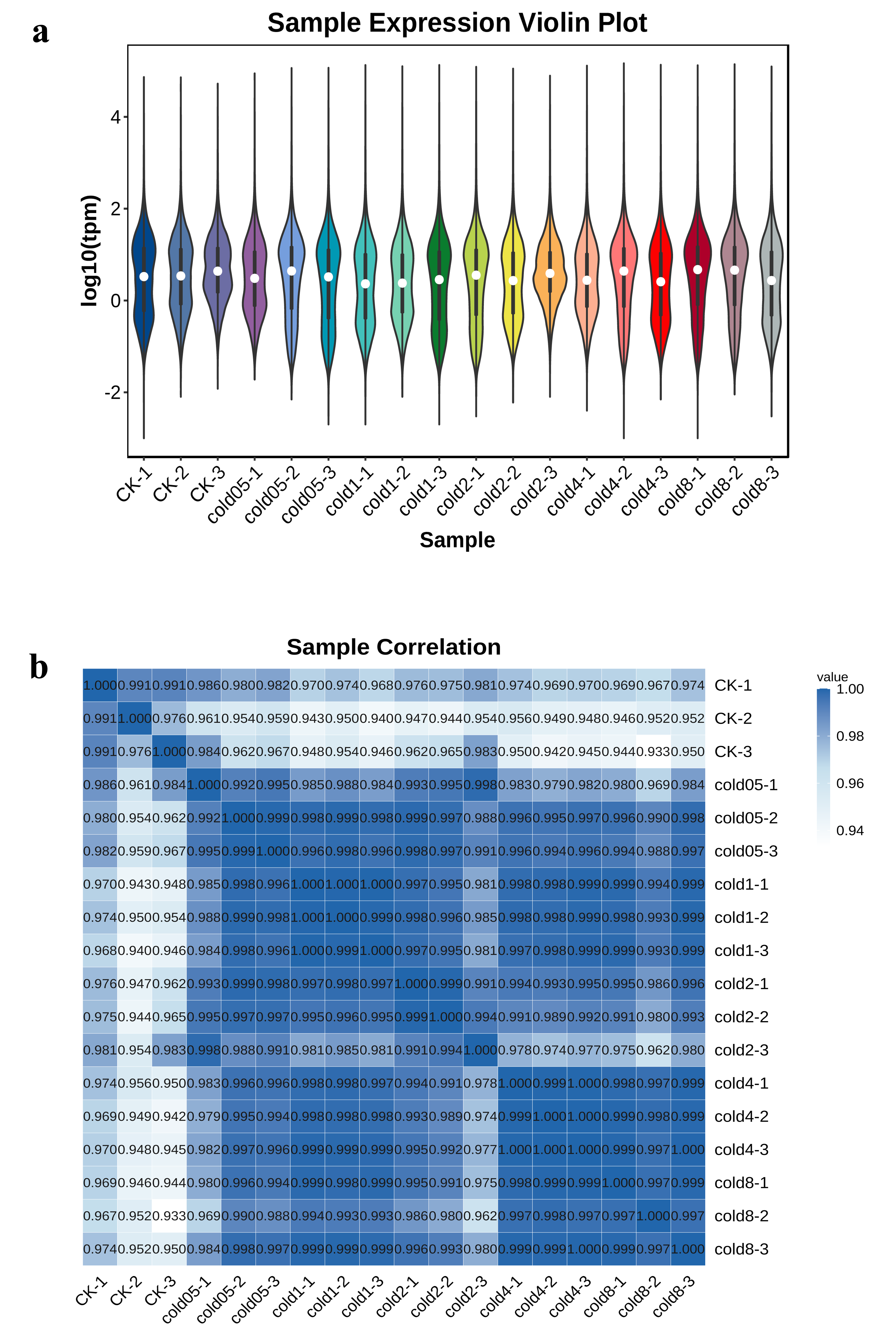

Supplement: Supplementary file 1 [file plants-15-01628-s001.zip › Supplemental Figure S2.tif]

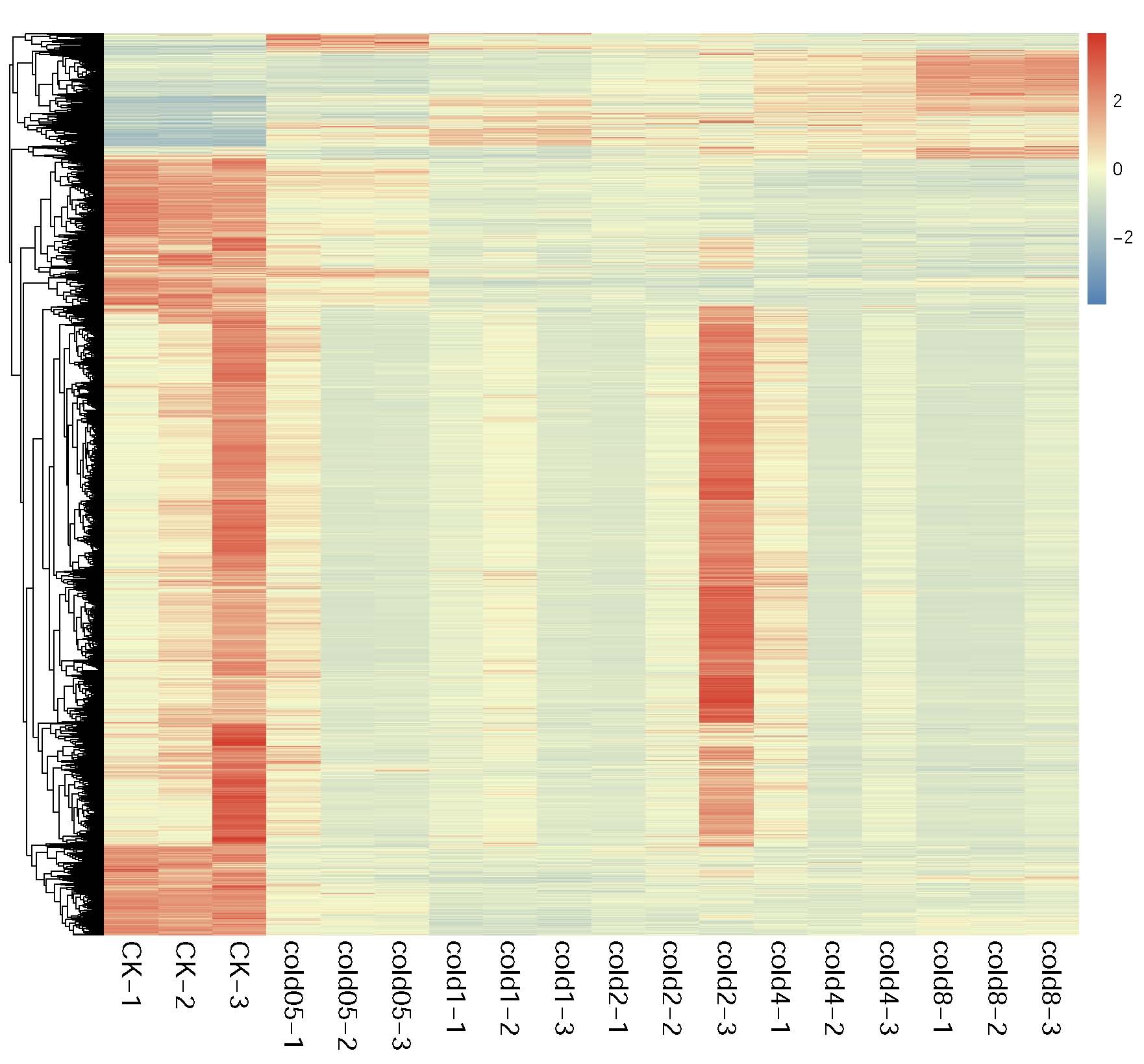

Supplement: Supplementary file 1 [file plants-15-01628-s001.zip › Supplemental Figure S3.png]

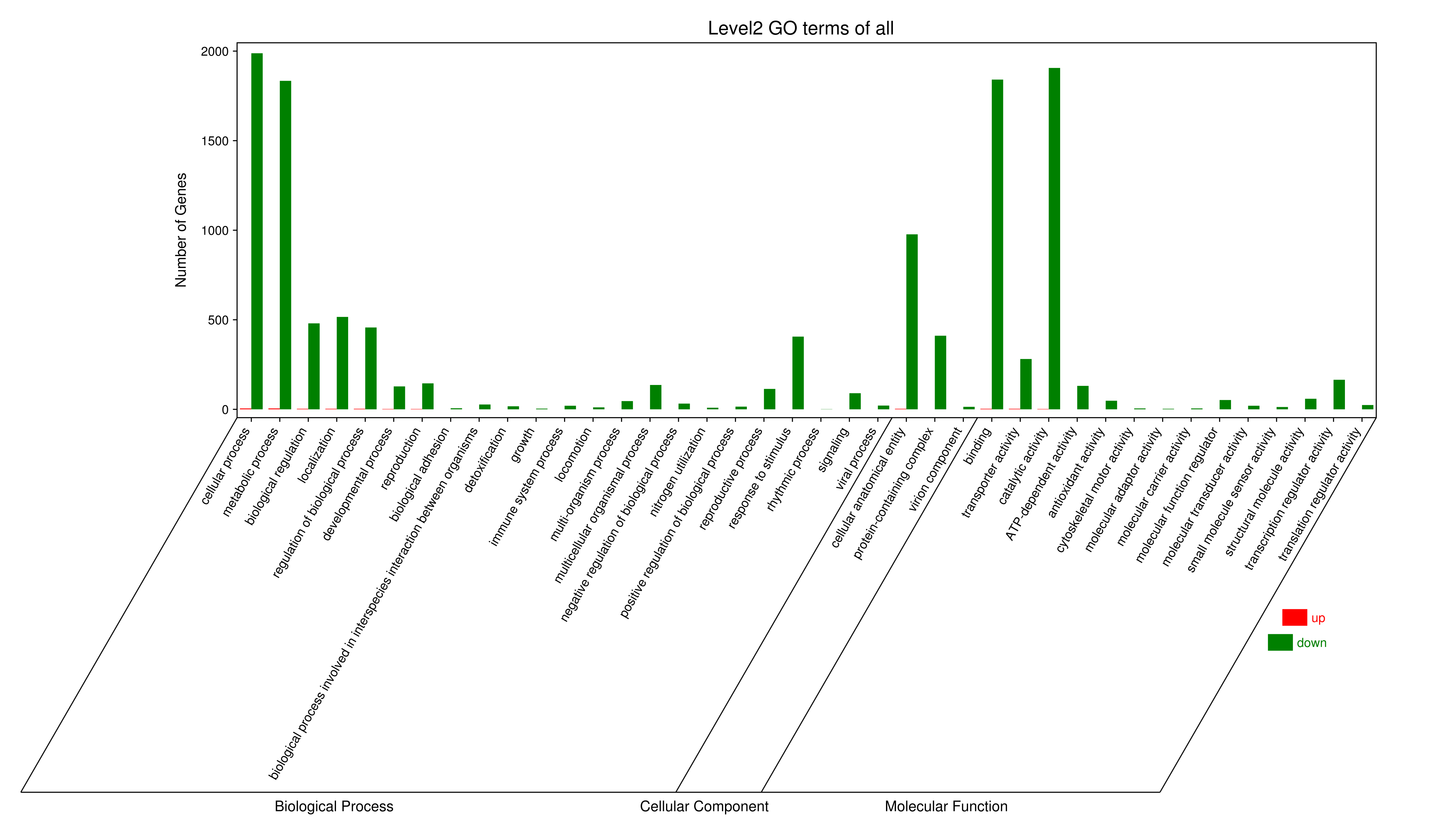

Supplement: Supplementary file 1 [file plants-15-01628-s001.zip › Supplemental Figure S4.png]

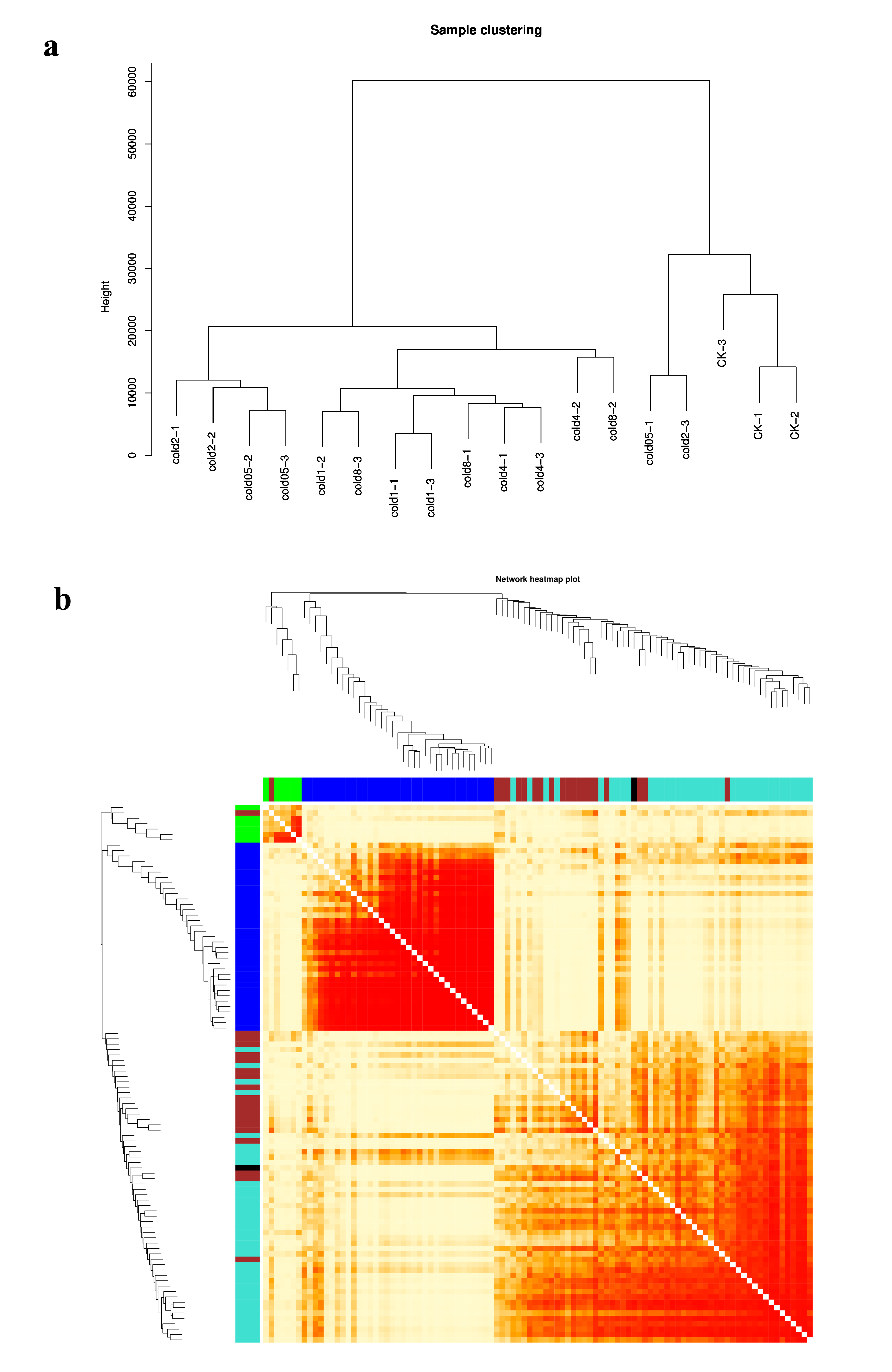

Supplement: Supplementary file 1 [file plants-15-01628-s001.zip › Supplemental Figure S5.tif]

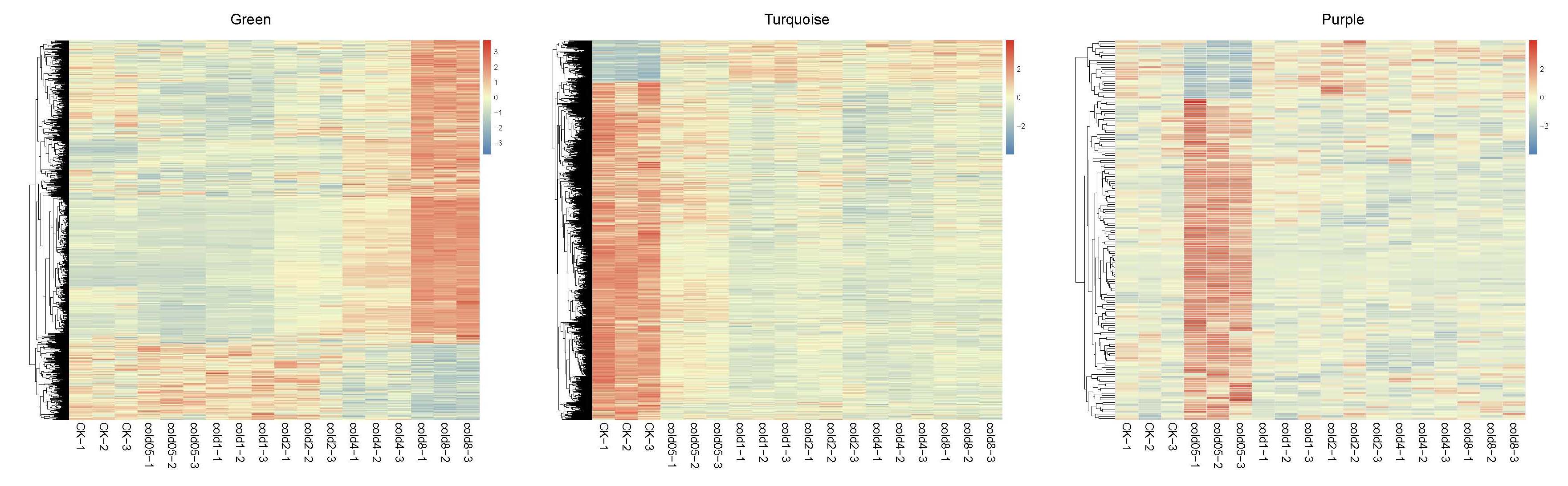

Supplement: Supplementary file 1 [file plants-15-01628-s001.zip › Supplemental Figure S6.png]

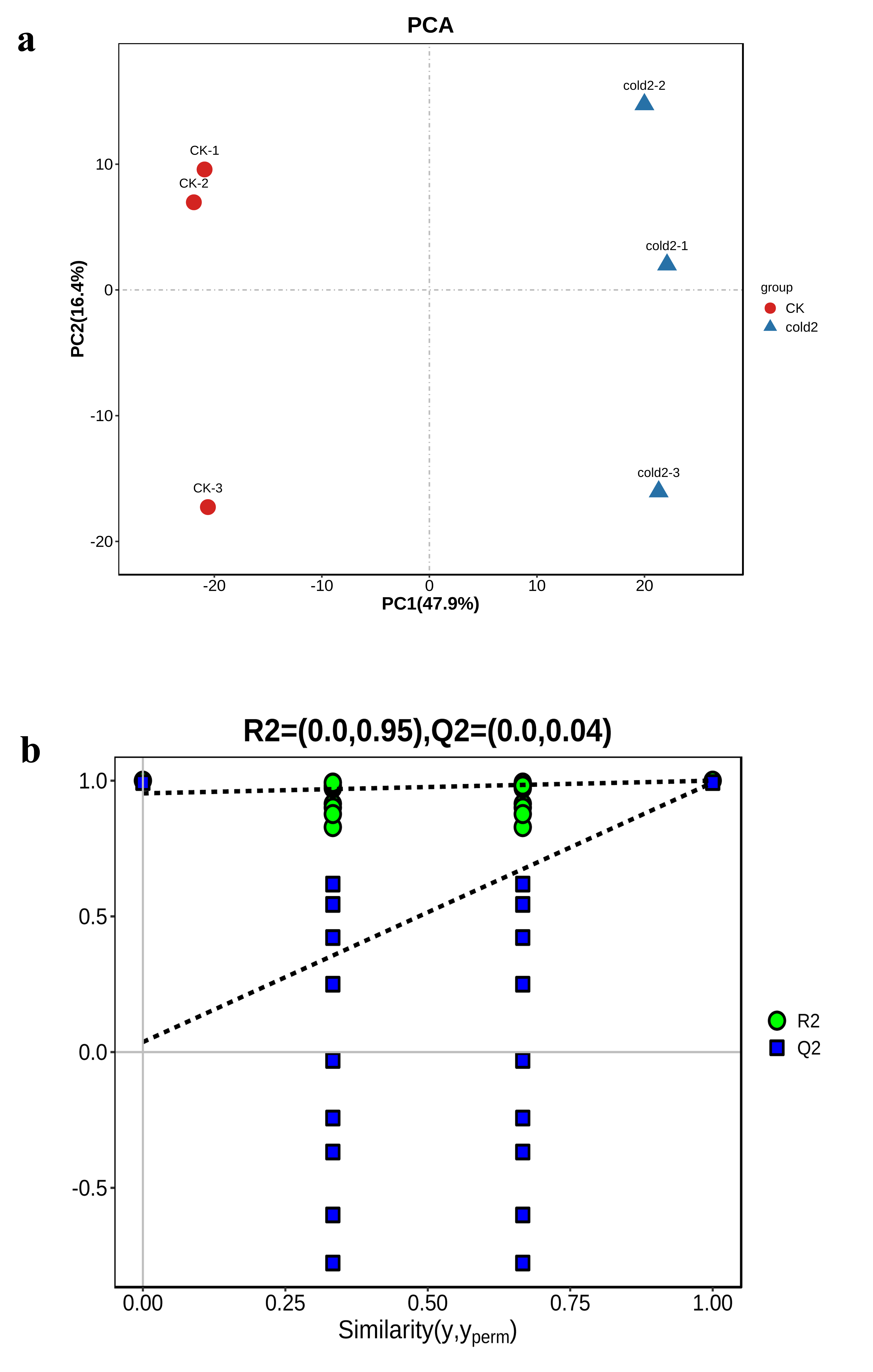

Supplement: Supplementary file 1 [file plants-15-01628-s001.zip › Supplemental Figure S7.tif]

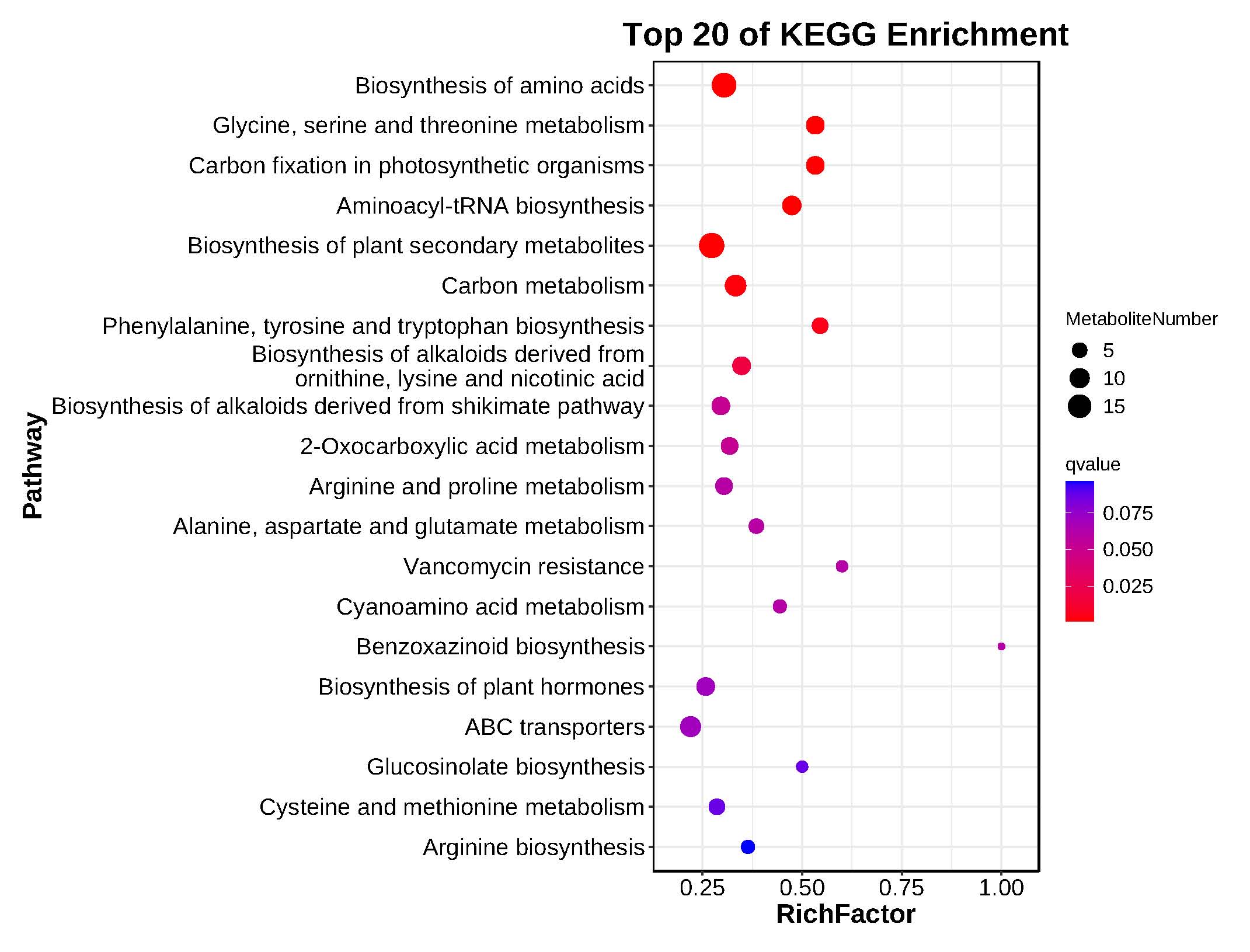

Supplement: Supplementary file 1 [file plants-15-01628-s001.zip › Supplemental Figure S8.png]
